# Wearable Sensors to Aid Rehabilitation Following Total Knee Arthroplasty: Experiences of Trial Participants

**DOI:** 10.7759/cureus.80752

**Published:** 2025-03-18

**Authors:** Samuel W King, Lara S Chapman, Tracey J Smith, Jeya Palan, Hemant Pandit

**Affiliations:** 1 Trauma and Orthopaedics, Leeds Institute of Rheumatic and Musculoskeletal Medicine, University of Leeds, Leeds, GBR; 2 Trauma and Orthopaedics, Leeds Teaching Hospitals NHS Trust, Leeds, GBR; 3 Podiatry, Leeds Institute of Rheumatic and Musculoskeletal Medicine, University of Leeds, Leeds, GBR

**Keywords:** participant experience, remote rehabilitation, total joint replacement, total knee replacement, wearable sensors

## Abstract

Background

Physiotherapy is key to satisfactory outcomes following total knee arthroplasty (TKA). However, financial constraints have necessitated a reduction in face-to-face post-operative outpatient physiotherapy sessions. Wearable sensors have been used for remote rehabilitation but with variable outcomes and adherence. In addition, patient experiences and views about the use of wearable sensors are rarely contextualised.

Methods

A single-centre prospective randomised controlled trial (RCT) is ongoing in the UK to assess the clinical and cost-effectiveness of wearable sensors in patients undergoing TKA. Participants of the accelerometry and rehabilitation after knee replacement (ARK) study were interviewed during a six-month follow-up clinic to explore trial experiences and suggestions for improvement. Responses were coded independently by two investigators using directed content analysis and a pre-determined framework of categories. Results were compared until consensus was reached. A summative approach was undertaken to analyse the resulting codes by frequency.

Results

Responses were recorded from 114 participants recruited in the ARK RCT: 62 from the intervention arm and 52 from the standard care arm. Common patient experience topics related to research involvement, wearable technology, rehabilitation support and communication, exercises, and general outcome. Areas for improvement included exercises, technology, questionnaires, and rehabilitation support and communication.

Conclusions

This study adds an important dimension to an RCT and provides direction for improvement. Research in both wearable sensors and the field of orthopaedics is often only quantitative in nature, and qualitative research provides a useful and unique dimension to these areas. Future work should further explore qualitative data in these fields.

## Introduction

Total knee arthroplasty (TKA) is an increasingly popular definitive management of end-stage arthritis. Demand continues to escalate globally as populations age and quality of life expectations increase [1,2]. TKA represents a large expenditure in most healthcare systems. A major portion of this relates to rehabilitation and is estimated to cost $500 million annually in the US alone [3,4]. There is no consensus regarding the post-operative rehabilitation protocols to be used, although enhanced recovery protocols are known to reduce length of stay [5,6].

Face-to-face outpatient physiotherapy improves objective functional outcomes and subjective patient-reported outcomes but increases costs [7,8]. In public healthcare systems, greater emphasis is now placed on self-directed outpatient physiotherapy due to financial constraints, but adherence rates with these can be as low as 30% [9]. A concomitant rise in the use of technology to assist this has followed [10]. Wearable sensors have the potential to allow remote monitoring of patient rehabilitation in the outpatient setting, aiding in adherence and allowing bespoke physiotherapy and early detection of complications [11]. Several studies in the literature have reported on wearable sensor use in remote rehabilitation after TKA. Some studies demonstrated similar outcomes where wearable sensors were used as an alternative to face-to-face therapy [12-14], while others identified an improvement when these were used in addition to standard post-operative rehabilitation [15-19].

A major factor in the success of remotely monitored rehabilitation continues to be adherence. The definition of satisfactory adherence varies across studies; it can relate to the attainment of goals [18], the rate of device usage [20], or self-reporting rates [19]. When reported, adherence rates in wearable sensor studies vary between 37% and 65% [12,15,16,18-20]. Research in this area has universally reported quantitative outcomes such as range of motion, physical activity, complications, and patient-reported outcome measures (PROMs). However, studies have rarely reported qualitative data, such as the experiences of trial participants or factors that may affect adherence. Those that have are relatively small studies with 25 or fewer participants [20,21]. A lack of understanding of underlying factors affecting adherence will continue to limit the applications of wearable sensors.

This paper investigates the experiences among trial participants in a randomised controlled trial (RCT) of wearable knee sensors to remotely monitoring. It also aims to understand and rationalise suggested improvements by the same participants. This is with the overall aim to assist in understanding factors affecting adherence and successful outcomes in the use of wearable sensors.

## Materials and methods

**Figure 1:**
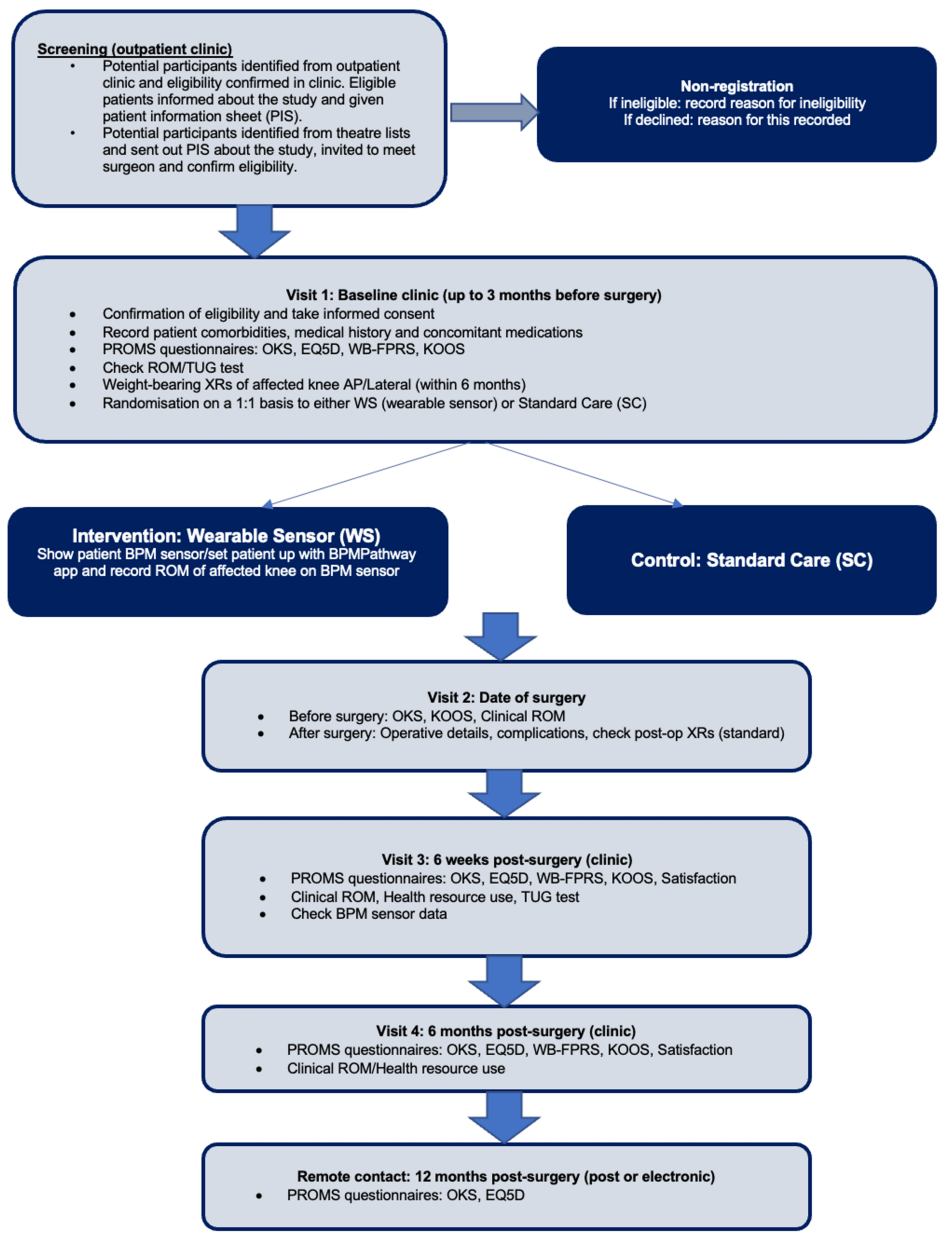
Recruitment and study timeline process OKS: Oxford knee score; EQ5D: EuroQuol-5 dimensions; WB-FPRS: Wong-Baker FACES Pain Rating Scale; KOOS: knee and osteoarthritis outcome score; ROM: range of motion; TUG: timed up and go test; PROMS: patient-reported outcome measures; XR: X-ray; AP: anteroposterior

The accelerometry and rehabilitation after knee replacement (ARK) study was registered in January 2022 and is a single-centre RCT (ClinicalTrials.gov Identifier: NCT05412940). The study recruitment and timeline process is available in Figure 1. Appropriate national research ethics committee (REC) approval and local institutional permission were granted prior to the start of the study. All participants provided informed consent. Eligible patients were those awaiting primary total knee replacement, aged 18 years of age or over with the ability to work with smart devices, in possession of an internet connection in their own home (mobile internet minimum 3G or Wi-Fi), and in possession of their own smart device compatible with the intervention software. The exclusion criteria were patients who were unwilling or mentally and/or physically unable to adhere to study procedures; had cognitive impairment which would prevent them from using the wearable device or mobile application; had previous joint arthroplasty in the same knee; had surgical treatment of the involved knee within the past six months (excluding arthroscopy); had previous orthopaedic surgery for trauma or arthritis of the knee joint (such as a previous fracture fixation or osteotomy); had active cancer (currently diagnosed and under treatment); unable to complete all trial procedures (e.g. attend follow-up visits, complete questionnaires); and were unable to provide informed consent.

In this study, control participants received the standard pre-operative and post-operative physiotherapy care: self-directed outpatient pre-operative exercises for up to 12 weeks followed by face-to-face physiotherapy during their peri-operative inpatient stay. This was followed by further self-directed outpatient rehabilitation exercises with a follow-up telephone call with the outpatient physiotherapy team to ensure adequate progress. Participants in the intervention group received the standard physiotherapy care and additionally were furnished with a wearable BPMpathway knee sensor (B. Braun Holding GmbH & Co. KG, Melsungen, Germany). This was worn during self-directed exercises to allow remote monitoring of patient rehabilitation. The sensor is connected to the participant’s smart device, which uploads data to the cloud at the end of the exercise session. The trial physiotherapist had access to this information and a live two-way messaging facility with the participant. The trial team could monitor the participant's pain score, range of motion, and frequency of exercises via the application and communicate with them when required. The sensor was used up until six months post-surgery. All participants were reviewed at follow-up clinics at six weeks and six months. A postal questionnaire was sent out at 12 months. All participants were given the contact details of the trial physiotherapist.

To enhance understanding of the experiences of trial participants, a trial physiotherapist asked two open-ended questions designed to ascertain information on the overall perception of the trial. It was made clear that this related to their experiences of the trial itself, their pre- and post-operative physiotherapy and (if applicable) the wearable sensor. The first 'Experiences' question was neutral and intended to be interpreted by the participant as they saw appropriate: 'What has your experience of the ARK study been so far?'. The second 'Improvements' question was intended to provide information to complement the trial’s quantitative findings by adding another dimension to responses: 'What (if anything) could be improved?'. These questions were asked at the routine six-month follow-up clinic and recorded verbatim immediately after each appointment. The six-month follow-up appointment was chosen as a time point as this was the end of the wearable sensor use in the intervention arm and the point at which the primary end point was measured in both arms.

Participants’ open-ended responses to questions relating to experiences were analysed using directed content analysis, as described by Hsieh and Shannon [22]. Initial categories were produced based on previous findings affecting experience and adherence [21], in addition to conversations with trial participants during follow-up clinics [21]. Data were coded by one researcher (SK) using this pre-determined framework; any data that could not be coded were identified and analysed to determine if they represented a new category or sub-category. A second coder (LSC) independently coded the open-ended responses. Coding discrepancies were discussed by both researchers, and codes were renamed until consensus was reached.

A summative approach was then undertaken to analyse the resulting codes. The frequency of mentions of each of the codes was recorded separately for standard care and intervention groups for each of the two questions. Frequencies of codes were counted by mention rather than by participant, i.e. a single participant response could count towards the frequency of multiple difference codes.

## Results

Responses were recorded from a total of 114 participants: 62 participants in the intervention arm and 52 in the standard care arm (22 male, 30 female). The baseline demographics and assessments are listed in Table 1. There were no major complications or re-operations in either group.

**Table 1:** Baseline demographics and assessments SD: standard deviation; BMI: body mass index; ASA grade: American Society of Anaesthesiologists grade; TUG: timed up and go; OKS: Oxford knee score

	Standard care	Wearable sensor
N	52	62
Age, mean (SD)	65.7 (8.24)	67.1 (7.91)
Gender (female %)	57.6%	58.1%
BMI, mean (SD)	32.4 (5.40)	32.1 (5.99)
ASA grade, mean (SD)	2.4 (0.59)	2.31 (0.56)
TUG time, mean (SD)	16.5 (23.97)	14.63 (13.27)
Knee flexion, mean (SD)	109.2 (15.26)	106.39 (11.84)
OKS, mean (SD)	17.2 (7.67)	19.02 (6.93)

Response coding

Responses relating to the 'Experiences' question ('What has your experience of the ARK study been so far?') were coded using three pre-determined categories: 'Exercises', 'Technology', and 'Rehabilitation support and communication'. Three new categories ('Research involvement', 'Outcome', and 'Nothing') were constructed during the initial analysis of responses. 'No response' was also added for participants who did not provide any relevant input. An overview of categories and codes is presented in Table 2.

**Table 2:** Experiences codes Categories and codes used to analyse responses to the question: 'What has your experience of the ARK study been so far?'

Category	Code
Research involvement	Enjoyment
	Altruism
	Non-beneficial
	General: positive
	Questionnaire negativity
Technology	Application issues
	Sensor issues
	Connectivity issues
	General reliability issues
	Ease
	Enjoyment
Rehabilitation support and communication	Trial-positive
	Trial-additive
	Institutional outpatient-negative
	Institutional inpatient-negative
	Institutional outpatient-positive
Exercises	Positive
	Incentive-motivate
	Incentive-self-monitor
	Difficulty
Outcome	Positive
Nothing	Nothing
No response	No response

Responses relating to the 'Improvements' question ('What (if anything) could be improved?') were coded using three pre-determined categories: 'Exercises', 'Technology', and 'Rehabilitation support and communication'. Two new categories ('Questionnaires' and 'No improvement required') were constructed during the initial analysis of responses. 'No response' was also added for participants who did not provide any relevant input. An overview of categories and codes is presented in Table 3.

**Table 3:** Improvements codes Categories and codes used to analyse responses to the question: 'What (if anything) could be improved?'

Category	Code
Exercises	Amend
	Additional
Technology	Software
	General
	Sensor reliability
	Application
	Manufacturer support
	Manufacturer instructions
	Accessibility
Questionnaires	Relevance
	Length
	Accessibility
Rehabilitation support and communication	Institutional
No improvement required	No improvement required

Trial participant experiences

**Figure 2:**
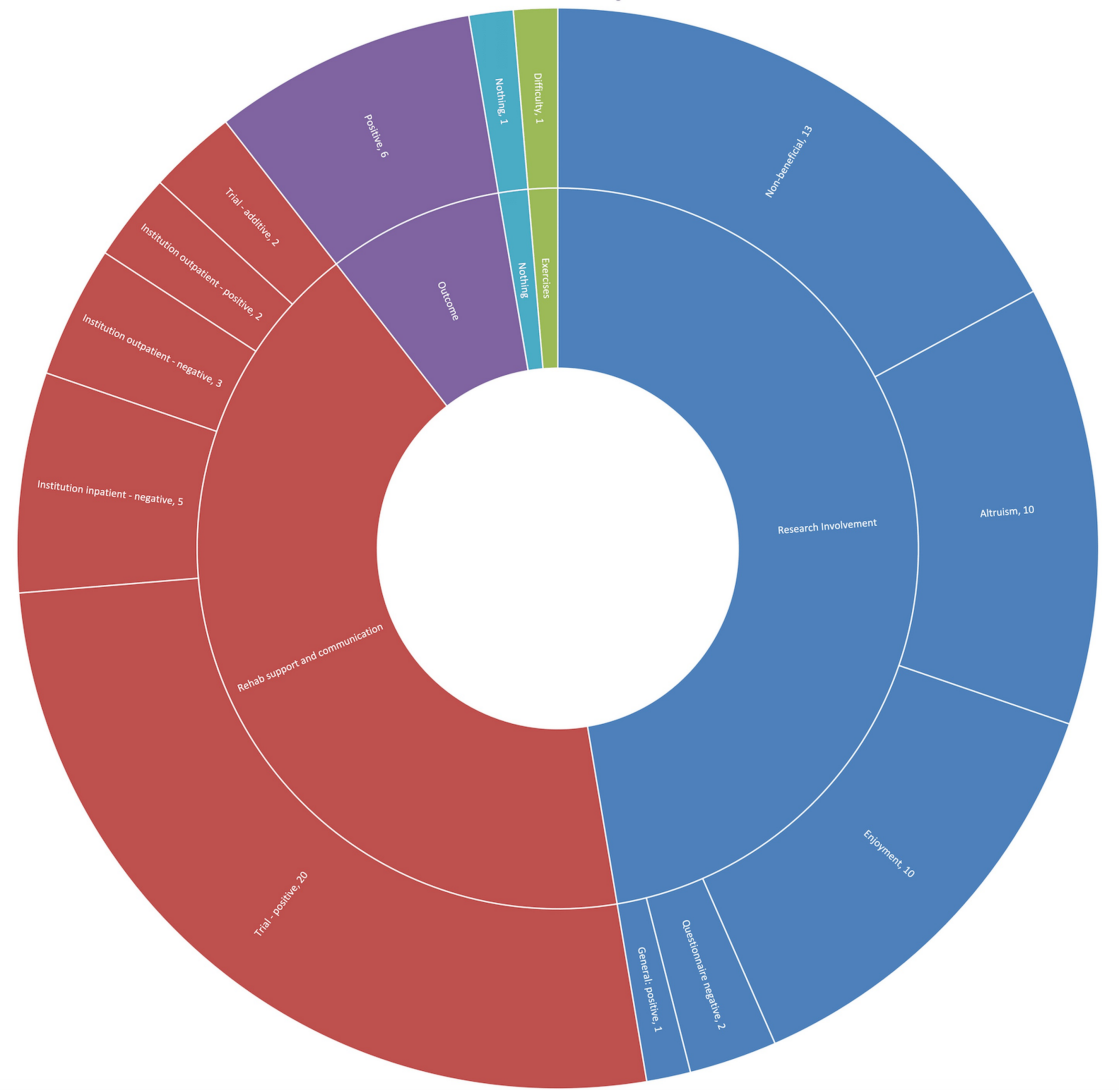
Standard care experiences ARK: accelerometry and rehabilitation after knee replacement A sunburst chart detailing the experiences of standard care participants in the ARK study

Standard care trial participant responses most frequently mentioned research involvement, with 36 mentions in total. Responses are visually displayed in Figure 2. Of these, 10 responses included a mention of enjoying being part of a study, while a further 10 had an altruistic component relating to helping future TKA patients. Thirteen of these responses were negative in nature, relating to feeling they were not personally benefiting from the study, for example, 'Didn’t feel like being part of a study apart from answering the questionnaires'. The next most common topic was around rehabilitation support and communication, mentioned in 32 responses. Of these, 22 felt that they had benefited in some way by having contact with the physiotherapist in the trial. Eight responses were in some way negative on the topic of the institutional physiotherapy they received, for example, 'Great access to extra Physio support when needed, not just a rubbish phone call'. Of these eight, five related to inpatient care and three to outpatient care. Two responses relating to institutional physiotherapy were positive. Meanwhile, six responses were positive about their overall outcome.

**Figure 3:**
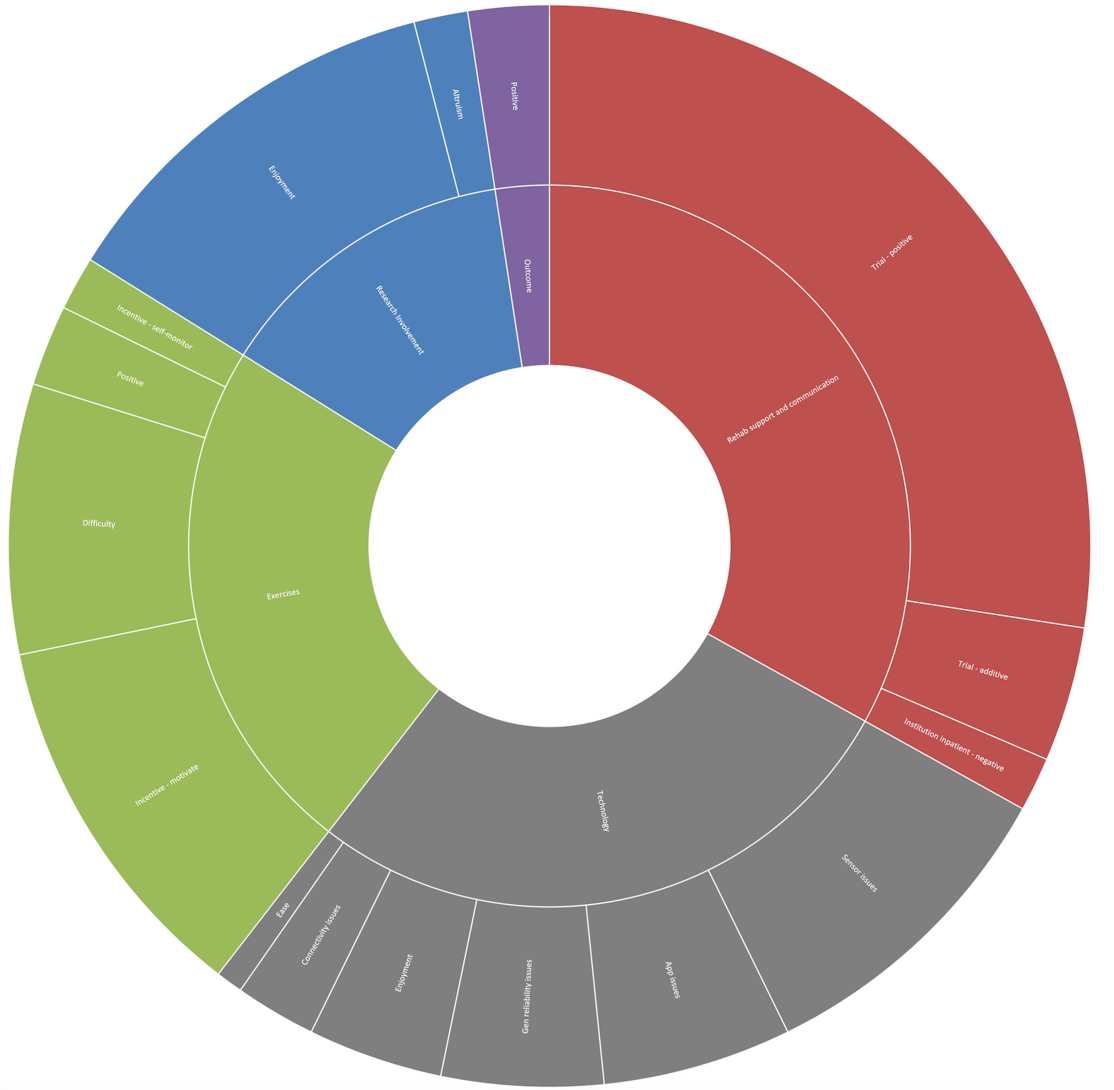
Wearable sensor experiences ARK: accelerometry and rehabilitation after knee replacement A sunburst chart detailing the experiences of wearable sensor participants in the ARK study

Participants assigned to the intervention arm most frequently responded relating to rehabilitation support and communication, with 41 responses relating to this category. Responses are visually displayed in Figure 3. Thirty-nine of these responses related to benefits from physiotherapy relating to the study (positive or additive), while two responses negatively related to institutional inpatient physiotherapy care. A large proportion of the responses related to the technology utilised (34). Six respondents found the technology easy or enjoyable, but most responses related to difficulties, either with the application (7), the wearable sensor (12), general reliability (6), or connectivity (3). The next most common category discussed was related to the exercises provided; 29 responses referred to this category. Sixteen responses identified that the exercises and sensor provided were an incentive, while three responses were generally positive about the experiences. Ten found the exercises provided difficult in some way. Fifteen responses referred to research involvement as enjoyable, while a further two were happy to be helping future patients.

Trial participant-suggested improvements

**Figure 4:**
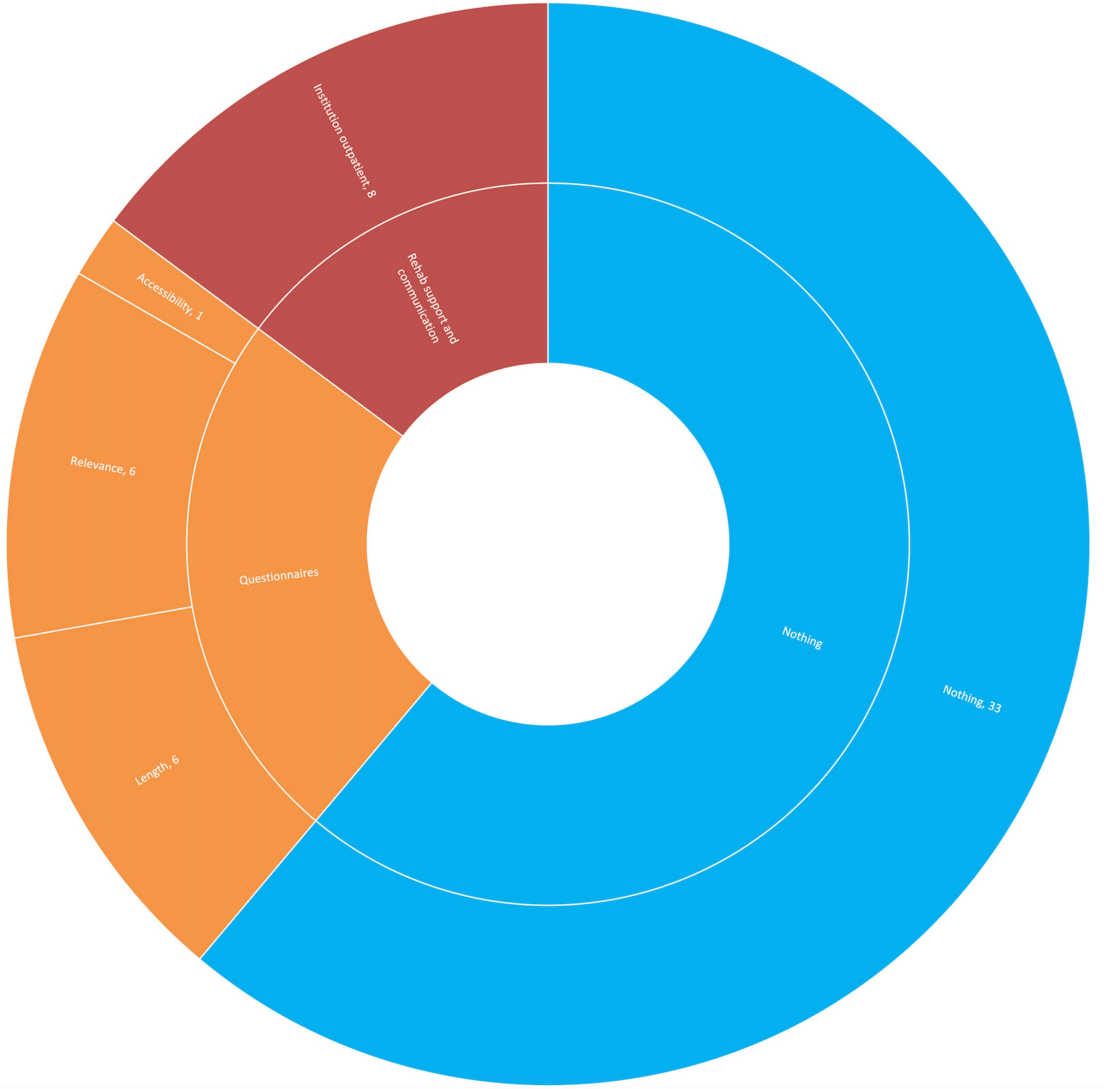
Standard care improvements ARK: accelerometry and rehabilitation after knee replacement A sunburst chart detailing the improvements suggested by standard care participants in the ARK study

Standard care participants mainly responded without suggestions for improvements. Responses are visually displayed in Figure 4. Thirteen responses related to the topic of questionnaires within the study: six related to participants feeling questionnaires should be made more relevant to them, six that they should be shorter, and one that they should be more accessible. Eight responses mentioned improvements in general institutional physiotherapy care.

**Figure 5:**
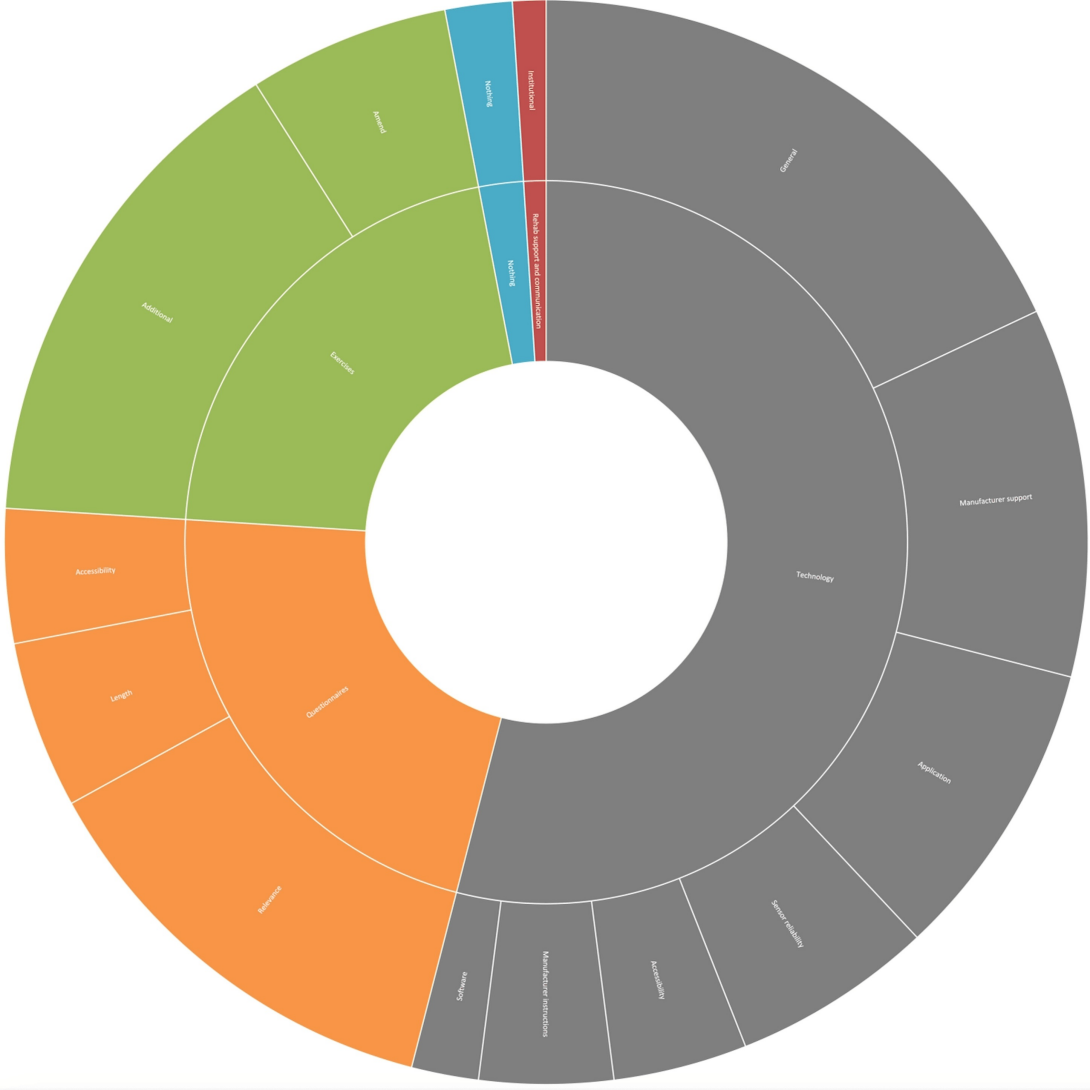
Wearable sensor improvements ARK: accelerometry and rehabilitation after knee replacement A sunburst chart detailing the improvements suggested by wearable sensor participants in the ARK study

Participants in the intervention arm responded with a larger number of suggestions. Responses are visually displayed in Figure 5. Fifty-four responses related to technology. Improvements were suggested for manufacturer support (11), mobile application or general software (11), sensor reliability (6), manufacturer instructions (4), and accessibility (4). Twenty-one responses referred to changes to the exercises: six regarding participants wanting these to be altered and 15 regarding a desire for more types of exercises. Twenty-two responses related to the trial questionnaires, with 13 about more relevant questionnaires, five that they were made shorter, and four that they were made more accessible. One response included a request for improved institutional physiotherapy, and two suggested no improvements.

## Discussion

This study enhances the understanding of the experiences of trial participants in a large single-centre RCT of wearable sensor use to aid remote monitoring of TKA patients rehabilitating before and after surgery. An interesting aspect of the feedback around participant experience related to rehabilitation and communication. Both groups reported positive experiences with trial-related physiotherapy (22 in the standard care group and 39 in the intervention group). This positive feedback related in part to the additional support received by the intervention arm participants because of the wearable sensor communication via the instant messenger in the associated mobile application but also because all trial participants were provided with the direct contact details of a trial physiotherapist. In a healthcare system where rehabilitation costs are high and routine face-to-face outpatient physiotherapy is often financially prohibitive [3,4], the importance of an experienced point of contact for assistance may often be overlooked. These findings reinforce its importance. The larger number of responses in the standard care group related to negative experiences of institutional physiotherapy and suggestions for its improvement compared with the intervention group, suggesting that the wearable technology was able to make up for shortfalls in this service.

The intervention group was generally positive with respect to the exercises utilised with their wearable sensors (19 out of 29 response mentions), and the suggestions for increased volume, numbers, and flexibility of exercises will be used in future iterations of the technology. Twenty-eight of the 34 responses from patients in the intervention arm regarding their experiences identified negative aspects of the wearable sensor. A major factor affecting the use of wearable sensors, in both research studies and clinical practice, is adherence. Adherence is variable in existing literature [12,18-20]. Adherence for self-directed exercise therapy is often poor, and this can be multifactorial [9]. These will be considered when interpreting the quantitative data and in conjunction with the 54 improvement suggestions, which will be invaluable in guiding improvements in the technology.

Several PROMs questionnaires were completed during this study. Patients in both arms responded negatively in relation to the length, accessibility, and relevance of these. The quantitative analysis of this study will be used to identify redundant or non-discriminating elements to reduce the questionnaire burden for future participants.

Finally, responses relating to involvement in the study were enlightening. Some participants’ responses in the standard care group identified altruistic reasons for taking part in the study and enjoyed taking part for this reason, while others mentioned disappointment about a less active role in the study and perceived a lack of benefit compared with participants in the intervention arm. This is a key finding for the involvement of people in research in general, and future studies should focus on ensuring that all participants feel that they are actively involved and contributing, especially those in the standard care arm.

Findings in this study were comparable to other, smaller studies in the literature. In studies of remote rehabilitation technology for knee replacement patients by Ramkumar et al. and Bell et al., participants also found the technology and extra support motivating and engaging, while their reported challenges also included the reliability of the technology and limitations of the exercise regime [20,21].

There are some potential limitations. The questions to participants were asked by a member of the trial, and this introduced potential bias in how the participants responded compared with an independent questioner. Responses were also recorded immediately after the clinic appointment rather than during, introducing the possibility of recall bias. Additionally, this work is from a single centre. Future studies are planned for a multi-centre trial of this technology with associated qualitative work as part of its assessment. Notwithstanding, this study provides an important insight into the experiences of trial participants in a field where this is often lacking.

## Conclusions

In a field of research where almost all focus is on quantitative data, experiential data is often overlooked. Research in both wearable sensors and the field of orthopaedics is often only quantitative in nature. Qualitative research provides a useful and unique dimension to these areas. These responses from trial participants in relation to their experiences and suggestions for improvements are invaluable to develop wearable technology and improve the quality of and engagement in future research. Future work in this field should further explore the use of qualitative data.
